# Concurrent follicular lymphoma and follicular dendritic cell sarcoma: composite neoplasm or transdifferentiation?

**DOI:** 10.1007/s12308-026-00736-z

**Published:** 2026-09-26

**Authors:** Mahsa Hajizadeh, Jinbo Fan, Jeffrey W. Craig, Ifeyinwa E. Obiorah

**Affiliations:** Department of Pathology, University of Virginia Health, Charlottesville, VA USA

**Keywords:** Follicular dendritic cell sarcoma, Follicular lymphoma, Composite lymphoma, Transdifferentiation, Fluorescence in situ hybridization

## Abstract

Follicular dendritic cell sarcoma (FDCS) is a rare neoplasm, and its synchronous occurrence with follicular lymphoma (FL) has not been reported. Whether composite tumors arise independently or through clonal evolution remains unclear. A 52-year-old woman presented with night sweats, weight loss, and an obstructing hepatic flexure colonic mass with mesenteric disease. Biopsy showed two tumor populations: an atypical B cell infiltrate positive for CD20, CD10, focal BCL6, and weak/focal MUM1, with kappa predominance and Ki-67 of 20–50%, consistent with follicular large B cell lymphoma/FL grade 3B, and epithelioid-to-spindled cells expressing CD21, CD23, CD35, CXCL13, and D2-40 and lacking B cell and epithelial markers, supporting FDCS. Fluorescence in situ hybridization demonstrated shared *BCL6* rearrangements, *IGH* and *MYC* copy-number gains in both components, *IGH* rearrangement testing was negative, and next-generation sequencing identified alterations in *JAK1*, *APC*, *FGFR4*, *NOTCH3*, and *TSC1*. This case highlights biologic challenges in distinguishing transdifferentiation from synchronous neoplasms.

**Supplementary Information:**

The online version contains supplementary material available at https://doi.org/10.1007/s12308-026-00736-z.

## Introduction

Follicular dendritic cell sarcoma (FDCS) is a rare neoplasm showing morphologic and immunophenotypic differentiation toward follicular dendritic cells and represents a small fraction of nodal and extranodal tumors [1, 2]. The coexistence of B cell non-Hodgkin lymphoma with histiocytic or dendritic cell sarcoma is uncommon, and its pathobiology has gained increasing attention [3]. Molecular studies have shown that follicular lymphoma (FL) and associated histiocytic/dendritic cell neoplasms may be clonally related, often sharing identical immunoglobulin gene rearrangements and IGH::BCL2 translocations [4, 5]. These findings support lineage plasticity or transdifferentiation of the FL clone, accompanied by loss of B cell programming, including downregulation of PAX5, and acquisition of myeloid/dendritic transcriptional programs such as C/EBPβ and PU.1 [5, 6]. Although histiocytic sarcoma and interdigitating dendritic cell sarcoma are more frequently reported in association with FL, the synchronous presence of FL and FDCS within the same biopsy specimen is exceedingly rare [6]. We report a case of concurrent FL and FDCS identified in a single biopsy, highlighting the diagnostic challenges and potential pathobiologic implications of this unusual composite neoplasm.

## Clinical history

A 52-year-old woman presented with a 1-month history of night sweats and weight loss. She reported abdominal pressure but denied pain, hematochezia, or changes in bowel habits. Abdominal ultrasound revealed a solid right upper abdominal mass concerning lymphadenopathy. Subsequent CT abdomen and pelvis confirmed a heterogeneous right upper abdominal mass measuring 12.5 × 8.1 × 14.7 cm involving the hepatic flexure of the colon with obstruction and adjacent mesenteric adenopathy. A positron emission tomography/computed tomography (PET/CT) scan demonstrated a large hypermetabolic mass (Supplemental Fig. 1A) extending from the inferior hepatic margin/hepatic flexure to the lower right hemiabdomen, with heterogeneous FDG uptake and a maximum SUV of 19.3. A more discrete hypermetabolic focus along the superior medial aspect measured 3.5 × 2.9 cm with a maximum SUV of 9.7, favored to represent an associated mass or nodal conglomerate. No chest or neck adenopathy, hepatosplenomegaly, or distant disease was identified. Colonoscopy (Supplemental Fig. 1B) revealed a noncircumferential, nonbleeding polypoid mass at the hepatic flexure measuring 8 cm in length and 5 cm in diameter, partially obstructing the lumen. A biopsy was obtained from the hepatic flexure mass.

**Fig. 1:**
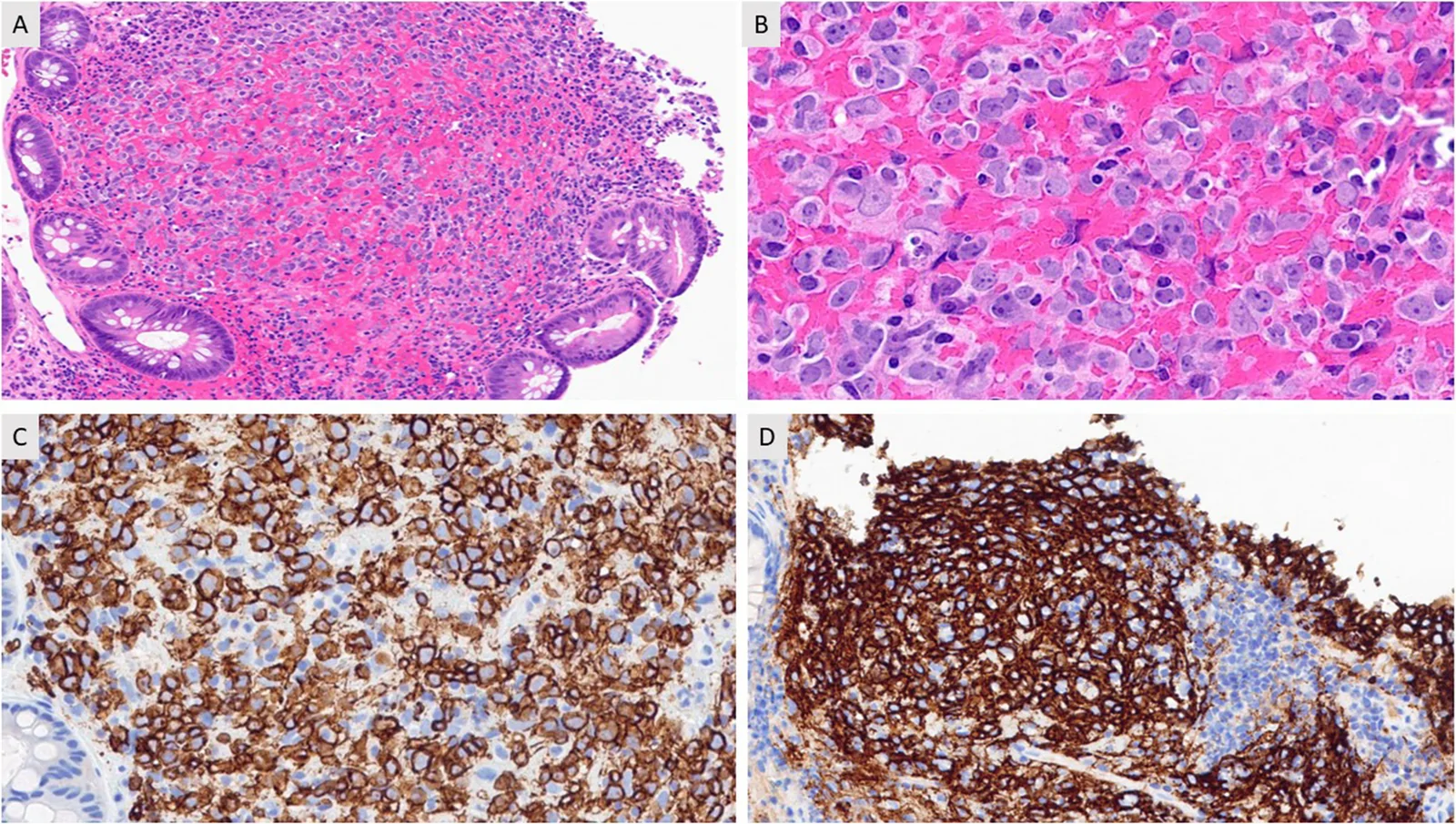
Pathologic features of the large B cell lymphoma involving the hepatic flexure colonic biopsy. **A** Hematoxylin and eosin (H&E) section shows focal involvement by a nodular neoplastic infiltrate (×10) composed of **B** large cells with irregular nuclear contours, vesicular chromatin, and variable nucleoli, admixed with fewer small lymphoid cells (×100). Immunohistochemical stains (40×) show that the atypical lymphoid cells are positive for **C** CD20. **D** CD23 highlights the associated follicular dendritic meshwork

## Materials and methods

### Histologic review

For the biopsies, hematoxylin and eosin (H&E) slides and immunohistochemical (IHC) stains were performed using routine protocols as previously described [7]. All negative and positive controls showed appropriate immunostaining.

### Fluorescence in situ hybridization and next-generation sequencing

The technical component of fluorescence in situ hybridization (FISH) was performed at Neogenomics per manufacturer’s protocol. For the next-generation sequencing, DNA extraction was performed on FFPE tissue from the colon biopsy using a QIAamp DNA FFPE tissue kit, per manufacturer specifications. PGDx elio™ tissue complete next-generation sequencing (NGS, 505 gene panel) analyses were performed using manufacturer’s recommended protocols. Analysis of PGDx data was performed by molecular pathology faculty according to professional guidelines [8, 9].

## Results

**Fig. 2:**
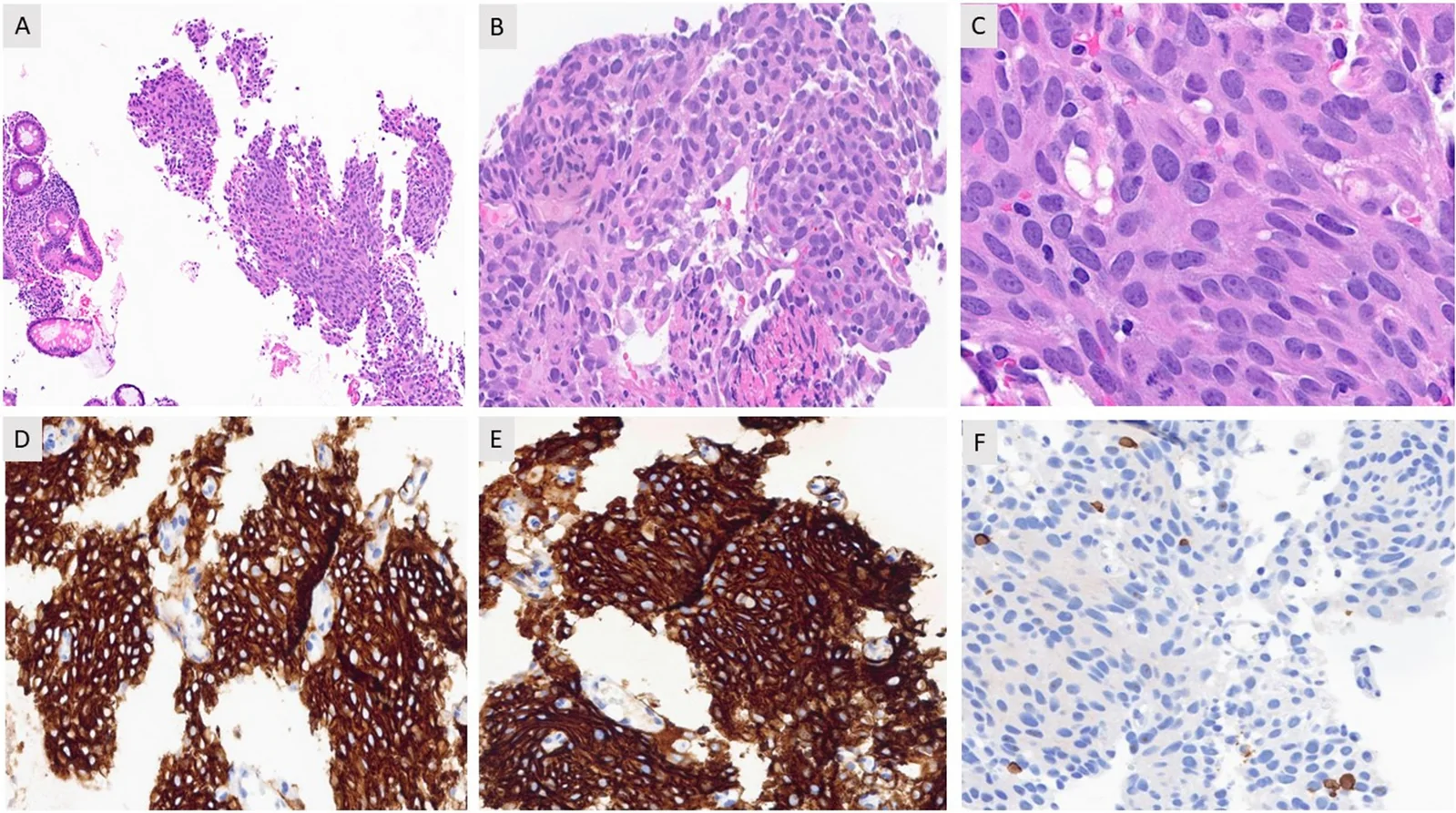
Pathologic features of follicular dendritic cell sarcoma involving the hepatic flexure colonic biopsy. **A** Hematoxylin and eosin (H&E) section shows involvement by a neoplastic infiltrate (×5) composed of **B** large epithelioid-to-spindled cells with irregular ovoid-to-reniform nuclei, dispersed chromatin, and abundant eosinophilic cytoplasm (**B** ×40, **C** ×100). Immunohistochemical stains (×40) show that the atypical cell population is positive for **D** CD21 and **E** CD23 and absence of **F** CD20 expression

Histologic sections showed two atypical tumor cell populations. The first (Fig. 1A, B) consisted of a nodular atypical lymphoid infiltrate composed of large cells with irregular nuclear contours, vesicular chromatin, and variable nucleoli, admixed with small lymphocytes and associated focal necrosis. The second consisted of multiple deposits of atypical epithelioid/spindled cells. By immunohistochemistry (IHC), the atypical B cells were positive for CD20 (Fig. 1C), CD10, focal BCL6, and weak/focal MUM1, with kappa predominance by RNAscope. They were negative for BCL2, c-MYC, cyclin D1, SOX11, and CD30. Ki-67 was 20–50% within this population. The nodular B cell areas were associated with well-formed follicular dendritic cell meshworks (Fig. 1D). CD21 and CD23 stains also highlighted the adjacent epithelioid and spindled cell population (Fig. 2), which showed weak BCL6 and MUM1 expression but was negative for CD20, BCL2, and CD10. EBV RNA was detected in only rare scattered cells. Overall, the findings were consistent with focal involvement by follicular large B cell lymphoma/FL grade 3B, with a concurrent atypical follicular dendritic cell neoplasm suggestive of FDCS.

**Fig. 3:**
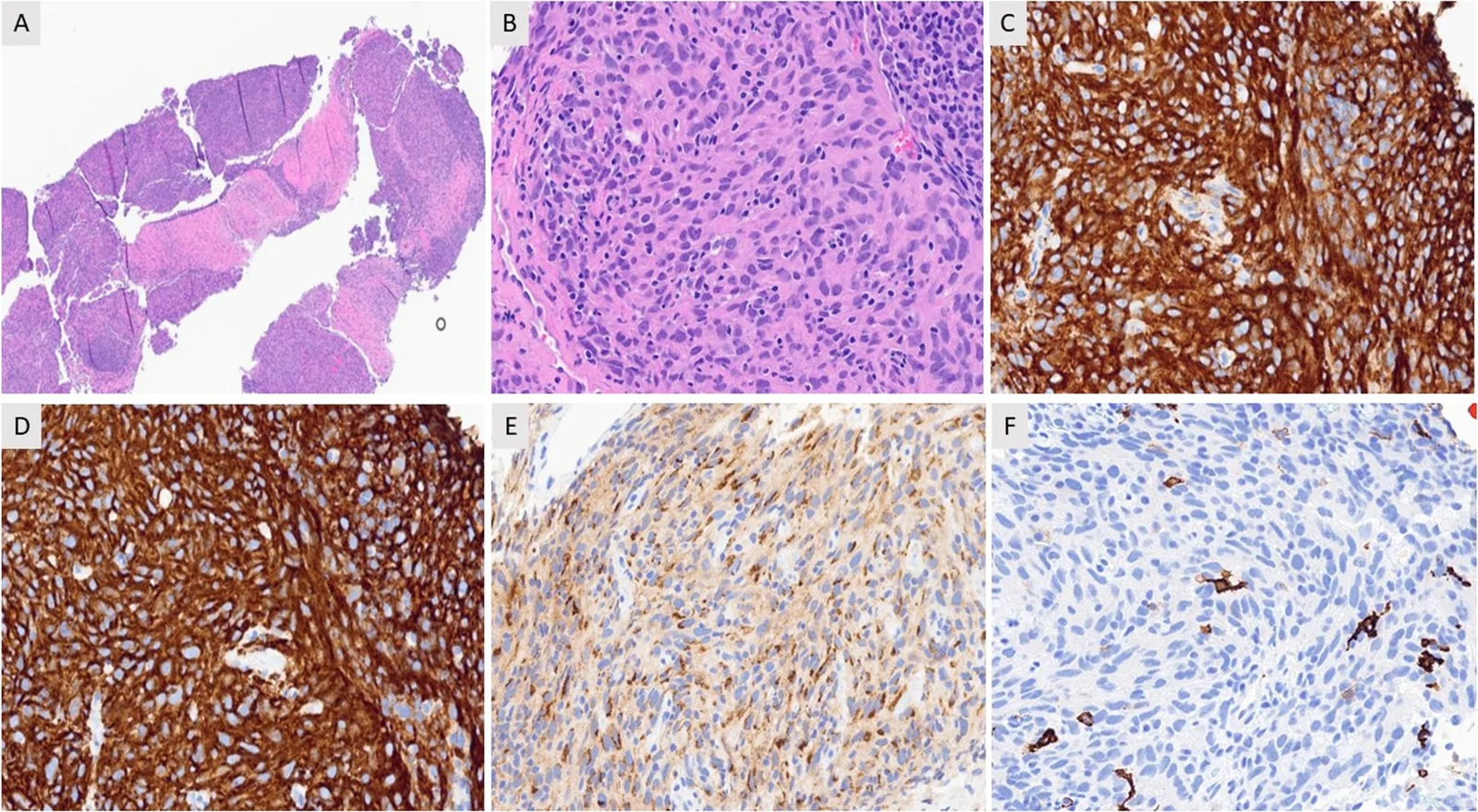
Pathologic features of follicular dendritic cell sarcoma involving the right abdominal mass biopsy. **A** Hematoxylin and eosin (H&E) section shows involvement by a neoplastic infiltrate (H&E ×5). **B** The infiltrate is composed of large epithelioid-to-spindled cells with irregular ovoid-to-reniform nuclei, dispersed chromatin, and abundant eosinophilic cytoplasm (H&E ×40). Immunohistochemical (IHC) stains (×40) show that the atypical lymphoid cells are positive for **C** CD21, **D** CD23, and **E** CXCL13 and negative for **F** CD20

A subsequent biopsy of the abdominal soft tissue mass (Fig. 3A, B) showed cohesive, vaguely nodular aggregates of large epithelioid-to-spindled cells with irregular ovoid-to-reniform nuclei, dispersed chromatin, and abundant eosinophilic cytoplasm with syncytial borders. By IHC, the atypical cells (Fig. 3C–F) were strongly positive for CD21, CD23, CD35, CXCL13, and D2-40, with weak/subset expression of BCL6, MUM1, and MYC, and equivocal CD4. They were negative for CD45, CD1a, CD68, CD163, T and B cell markers, CD10, CD30, CD43, BCL2, MPO, S100, and cytokeratin. Ki-67 was approximately 10–15%. EBV RNAscope highlighted scattered nonneoplastic cells and was negative in the tumor cells. The features were consistent with FDCS. No distinct abnormal B cell aggregates were identified in these sections.

**Fig. 4:**
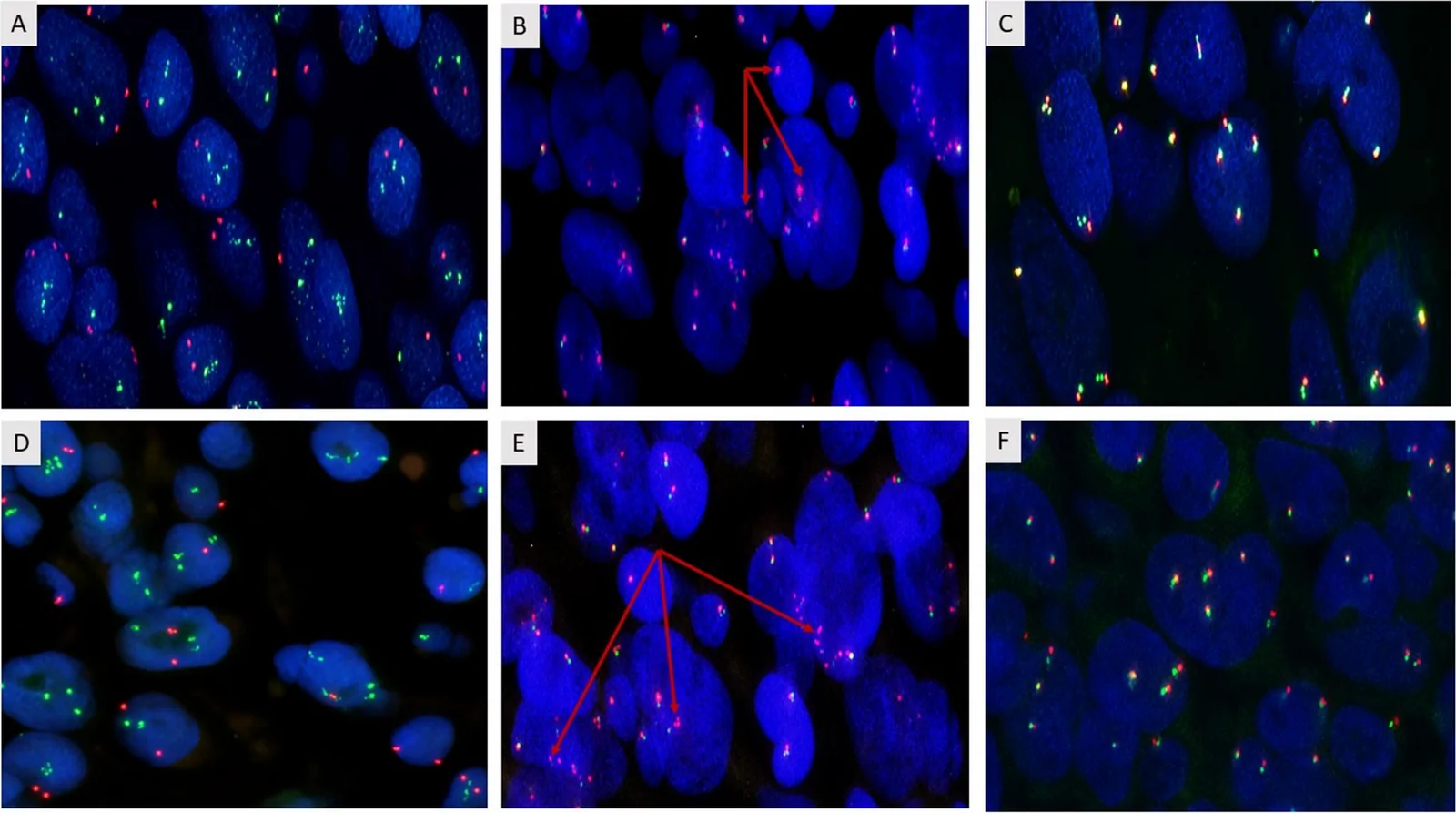
Fluorescence in situ hybridization (FISH) analysis of the follicular lymphoma (FL) and follicular dendritic cell sarcoma (FDCS) components. **A**–**C** FL component. **A** Dual-color, dual-fusion IGH/BCL2 FISH showing an atypical abnormal pattern (IGH—green, BCL2—red) (26% of nuclei), indicative of either *IGH* translocation with a partner other than *BCL2*, trisomy 14, or duplication 14q. **B** *BCL6* break-apart FISH demonstrating an abnormal signal pattern (in 42% of nuclei) with one isolated red signal (red arrows) from the rearranged allele (indicating separation of the 5′ probe from its 3′ counterpart, with loss of the corresponding green signal) suggestive of a variant or unbalanced rearrangement pattern. **C** *MYC* break-apart FISH showing gains of *MYC* (3–4 fusion (red-green or yellow) in 30% of nuclei). **D**–**F** FDCS component. **D** Dual-color, dual-fusion IGH/BCL2 FISH showing an atypical abnormal pattern suggestive of trisomy 14, *IGH* gene duplication, or an *IGH* rearrangement with a chromosome other than *BCL2* on chromosome 18 (in 24% of nuclei). **E** *BCL6* break-apart FISH demonstrating an abnormal signal pattern (in 42% of nuclei) similar to FL indicating a variant or unbalanced rearrangement pattern. **F** MYC break-apart FISH showing gains of *MYC* (in 56% of nuclei)

FISH (Fig. 4) performed separately on both the FL and FDCS cell populations of the initial hepatic flexure mass biopsy detected gains of *MYC* and *IGH* as well as partial deletion of 3′ *BCL6* which is compatible with either a variant or unbalanced *BCL6* rearrangement. Molecular studies performed on the colonic biopsy were negative for a clonal *IGH* gene rearrangement, whereas NGS (Table 1) detected an activating/gain-of-function mutation in the pseudokinase domain of *JAK1* as well as variants of uncertain significance involving *APC*, *FGFR4*, *NOTCH3*, and *TSC1*. The patient underwent a hemicolectomy and will be undergoing post resection chemotherapy.

**Table 1:** Sequencing analysis of the colonic biopsy

Gene	Alteration	Consequence	VAF (%)
APC	G2677D	Missense	48.8
FGFR4	E326D	Missense	46.9
JAK1	V658F	Missense	7
NOTCH3	R1851C	Missense	49.5
TSC1	R22W	Missense	42.7

## Discussion

The presence of both FL and FDCS in the patient’s initial hepatic flexure mass biopsy raises the possibility of either independent composite neoplasms or clonally related tumors arising through transdifferentiation. FL-to-FDCS transdifferentiation is rare and remains poorly understood. Although transdifferentiation has been described as lineage conversion of a mature B cell neoplasm into a histiocytic or dendritic cell sarcoma, the WHO fifth edition [10] reclassified FDCS as a stroma-derived neoplasm of lymphoid tissues, reflecting their mesenchymal rather than hematopoietic origin [11]. However, the International Consensus Classification [12] continues to classify these tumors within the category of histiocytic and dendritic cell neoplasms. Based on the available literature, no prior report of FL specifically occurring as either a composite or concurrent tumor with FDCS has been identified. FDCS is an uncommon malignant tumor that recapitulates the morphology and immunophenotype of follicular dendritic cells, a specialized mesenchymal stromal cell population that supports germinal center architecture and function [13]. Beyond the rare association with lymphoid neoplasms, 7–10% of cases occur in the setting of hyaline-vascular Castleman disease [14]. Genomic studies [2] have shown that FDCS frequently harbors loss-of-function alterations involving NF-κB regulatory genes, including *NFKBIA*, *CYLD*, *BIRC3*, *TRAF3*, and *TNFAIP*3, as well as alterations in cell cycle regulatory genes such as *CDKN2A*, *RB1*, and *TP53.* MYC structural abnormalities [15], including gains and rearrangements, have also been reported in approximately 36% of cases. In contrast, MAPK pathway mutations, which are characteristic of many histiocytic neoplasms, are uncommon in FDCS, supporting a distinct pathogenesis [15]. Interestingly, despite the mesenchymal origin of FDCS, clonal immunoglobulin gene rearrangements have been identified in a subset of tumors [16].

In our case, several findings support a possible clonal relationship. Both the FL and FDCS components showed gains of *MYC* and *IGH* as well as evidence of *BCL6* rearrangement. Although *MYC* alterations have been reported in both FL and sporadic FDCS [15], thereby raising the possibility of convergent evolution, the shared presence of three different chromosomal abnormalities is unlikely to occur independently in two spatially associated but clonally unrelated neoplasms. Interestingly, a clonal IGH gene rearrangement was absent in the colonic biopsy. This may reflect technical limitations, sampling variability, or tumor heterogeneity. Lack of diagnostic assays precluded the performance of IGK gene rearrangement studies. Notably, the *JAK1* mutation detected in our case is unique and previously undescribed in FDCS. Although the significance of the *TSC1* and *APC* variants described here remains uncertain, Schelbert et al. identified *TSC1* and *APC* mutations among the tumor suppressor gene alterations detected by targeted NGS analysis of 13 FDCS cases [2]. The described *APC* mutation in their study was newly acquired at recurrence while an initially detected *NFKBIA* mutation was lost, suggesting clonal evolution with *APC* mutation emerging as a potential driver during disease progression. Notably, *TSC1* is a critical regulator of dendritic cell development and function [17]. Mutations in *TSC1* may result in decreased stability or loss of TSC1, disrupting the TSC1-TSC2-mediated inhibition and causing constant activation of mTORC1 [18]. This process encourages cell growth, proliferation, and changes in metabolism.

Overall, the shared *MYC*, *IGH*, and *BCL6* alterations in both components suggest a possible clonal relationship between the FL and FDCS. These findings highlight the biological complexity of composite FL/FDCS and the need for cautious interpretation of shared cytogenetic abnormalities. Further studies involving additional cases are necessary to better understand the relationship and underlying mechanisms between these neoplasms.

## Supplementary Information

Below is the link to the electronic supplementary material.

ESM 1 (DOCX 1.42 MB)

## Data Availability

No datasets were generated or analyzed during the current study.
