# Supplementary material for: Concurrent follicular lymphoma and follicular dendritic cell sarcoma: composite neoplasm or transdifferentiation?
Source: J Hematop. 2026 Sep 26;19(1):60. doi: 10.1007/s12308-026-00736-z (PMC13615919; doi:10.1007/s12308-026-00736-z)
Supplement: Supplementary file 1 [file 12308_2026_736_MOESM1_ESM.docx]

Supplemental Figure

Supplemental Figure 1. Radiologic and endoscopic findings of the colonic mass.

(A) PET-CT image of the abdomen demonstrating a large, heterogeneous right upper abdominal mass, measuring 12.5 × 8.1 × 14.7 cm (orange crosshairs), extending from the inferior hepatic margin/hepatic flexure to the lower right hemiabdomen. (B) Colonoscopic view of the hepatic flexure reveals a large, lobulated, non-circumferential polypoid mass.
